# The *Staphylococcus aureus* Membrane Protein SA2056 Interacts with Peptidoglycan Synthesis Enzymes

**DOI:** 10.3390/antibiotics2010011

**Published:** 2013-01-22

**Authors:** Chantal Quiblier, Agnieszka Luczak-Kadlubowska, Esther Holdener, Daniela Alborn, Tanja Schneider, Imke Wiedemann, Mariana G. Pinho, Hans-Georg Sahl, Susanne Rohrer, Brigitte Berger-Bächi, Maria Magdalena Senn

**Affiliations:** 1 Institute of Medical Microbiology, University of Zurich / Gloriastrasse 32, 8006 Zurich, Switzerland; E-Mails: chantalquiblier@access.uzh.ch (C.Q.); esther.holdener@alumni.ethz.ch (E.H.); susanne_elisabeth.rohrer@alumni.ethz.ch (S.R.); bberger@imm.uzh.ch (B.B.-B.); 2 Centre of Quality Control in Microbiology / ul. Chelmska 30/34, 00-725 Warsaw, Poland; E-Mail: luczakowa@interia.pl; 3 AMSolutions, ul. Debowa 32, 05-123 Chotomow, Poland; 4 Institute for Medical Microbiology, Immunology and Parasitology, University of Bonn / Meckenheimer Allee 168, 53115 Bonn, Germany; E-Mails: dalborn@uni-bonn.de (D.A.); tanja@mibi03.meb.uni-bonn.de (T.S.); iwiedemann@medpharmaservice.de (I.W.); sahl@mibi03.meb.uni-bonn.de (H.-G.S.); 5 Instituto de Tecnologia Química e Biológica, Universidade Nova de Lisboa / Av. Da Républica (EAN), 2781-901 Oeiras, Portugal; E-Mail: mgpinho@itqb.unl.pt

**Keywords:** *Staphylococcus aureus*, RND protein, FemABX, PBP, peptidoglycan, bacterial two-hybrid system

## Abstract

The yet uncharacterized membrane protein SA2056 belongs to the ubiquitous RND (Resistance-Nodulation-cell Division) family of transmembrane efflux transporters. The *sa2056* gene is located downstream of *femX*, the gene encoding the essential, non-ribosomal peptidyl-transferase adding the first glycine in the staphylococcal cell wall pentaglycine interpeptide. Due to its proximity to and weak co-transcription with *femX*, we assumed that *sa2056* may somehow be involved in peptidoglycan synthesis. Specific antibodies against SA2056 showed that this protein is expressed during growth and present in the membrane fraction of cell preparations. Using a bacterial two hybrid system, SA2056 was shown to interact (i) with itself, (ii) with FemB, which adds glycines 4 and 5 to the peptidoglycan interpeptide and (iii) with the essential penicillin binding proteins, PBP1 and PBP2, required for cell division and incorporation of the peptidoglycan into the cell wall. Unexpectedly, deletion of *sa2056* led to no phenotype regarding growth, antibiotic resistances or cell morphology; nor did *sa2056* deletion in combination with *femB* inactivation alter β-lactam and lysostaphin sensitivity and resistance, respectively, pointing to possible redundancy in the cell wall synthesis pathway. These results suggest an accessory role of SA2056 in *S. aureus* peptidoglycan synthesis, broadening the range of biological functions of RND proteins.

## 1. Introduction

One of the most common nosocomial human pathogens, *Staphylococcus aureus* can cause a variety of hospital- and community-acquired infections and intoxications. Treatment of this Gram-positive bacterium has become difficult due to its ability to rapidly develop resistance against virtually all currently used antibiotics. Genes potentially involved in cell wall synthesis, a pathway unique to bacteria, may represent novel targets for the therapy of staphylococcal infections.

The main component of the bacterial cell wall is a three-dimensional peptidoglycan meshwork whose backbone consists of the alternating saccharides N-acetylglucosamine and N-acetylmuramic acid (MurNAc). The characteristic pentapeptide branching off the MurNAc unit is synthesized in *S. aureus* by three non-ribosomal peptidyl-transferases; FemABX. Using Gly-tRNA as donor and the peptidoglycan precursor lipid II as substrate, they add in a sequential fashion five glycines to form a characteristic pentaglycine interpeptide (Gly_5_) [1,2,3,4]. Cross-linking of adjacent peptidoglycan strands and anchoring of surface proteins, contributing to the virulence of *S. aureus*, occurs via this Gly_5_-interpeptide [5]. An incomplete Gly_5_ interpeptide leads to aberrant growth, requiring compensatory mutations to assure survival [6,7], while a complete lack is lethal [2]. Importantly, methicillin resistant *S. aureus* (MRSA) depend for high-level resistance on the correct formation of the peptidoglycan precursor, including a complete Gly_5_ chain [8,9,10]. After transport across the cellular membrane, the peptidoglycan precursor is incorporated into the existing cell wall by the PBPs (for *p*enicillin *b*inding *p*rotein), exoplasmic enzymes catalyzing transglycosylation of the sugar moiety and transpeptidation of the Gly_5_ chain. β-lactams, such as methicillin, inhibit the latter reaction by irreversibly binding to the active site of the transpeptidase domain. 

The *orf* down-stream of *femX, sa2056*, encodes a putative 114.7 kDa protein with 12 predicted transmembrane domains belonging to the resistance-nodulation-cell division (RND) family. RND proteins are ubiquitous and have diverse biological functions, ranging from multidrug exporters, such as AcrB in *Escherichia coli* to morphogen receptors in *Drosophila melanogaster*, as found for patched (Ptc) (reviewed in [11]). SA2056 is annotated as a hydrophobic/amphiphilic exporter-1 (HAE1) family protein (subclass 2.A.6.2 in the Transporter Classification database; TCDB [12]), with 93% of the SA2056 amino acid sequence matching AcrB/AcrD/AcrF family motifs (Kyoto Encyclopedia of Genes and Genomes database; KEGG [13]).

The genetic organization *femX*-*sa2056* is conserved among all published annotated staphylococcal species. Previous attempts to knock-out *sa2056* had been unsuccessful, and Northern blot analyses had indicated co-transcription of *femX* and *sa2056* [14], suggesting *sa2056* to be essential and to have a cell wall-related function associated with *femX*. 

Both *femX* and *sa2056* lie on the negative strand of the *S. aureus* chromosome and are separated by a 117 bp segment. Rho-independent transcription terminators are predicted by TransTermHP downstream of both *femX* and *sa2056* [15]. Apart from the promoter upstream of *femX*, the program softberry identified an additional putative promoter in the intergenic region between *femX* and *sa2056* [16]. Microarray analyses had shown *sa2056* to be slightly upregulated in response to daptomycin, peracetic acid and chlorination [17,18,19]. On the other hand, *sa2056* is downregulated by mupirocin and mitomycin and in a *graRS* and *clpP* mutant background [20,21,22]. These alterations are paralleled by *femX* only in the case of daptomycin or mupirocin challenge and in the *clpP* mutant, suggesting that transcription of *femX* and *sa2056* can occur simultaneously or autonomously, depending on the conditions. Interestingly, SA2056 was found to harbor single-nucleotide polymorphisms (SNPs) in an *in vitro* generated ceftobiprole-resistant *mecA*-negative COL variant [23]. In this strain, additional SNPs were present only in two other genes: in *pbp4* encoding the only low-molecular-weight PBP of *S. aureus*, PBP4 and in *gdpP*, influencing the levels of the second messenger c-di-AMP [24]. Both PBP4 and GdpP directly or indirectly play a role in cross-linking of peptidoglycan and β-lactam resistance [25,26,27,28,29,30], further supporting the hypothesis that also SA2056 could play a role in peptidoglycan synthesis.

In this study, analysis of the markerless *sa2056* knock-out mutant CQ33 [31] was extended to various growth and stress conditions. In addition, SA2056 was tested for interaction with peptidoglycan synthesis enzymes in a bacterial two hybrid system and in pull-down experiments. Although we could not find a phenotype for the mutant, we could show that SA2056 interacted with some of the FemABX factors and the PBPs, suggesting SA2056 to play a subsidiary role in peptidoglycan synthesis.

## 2. Results and Discussion

### 2.1. Expression of sa2056 During Growth

**Figure 1:**
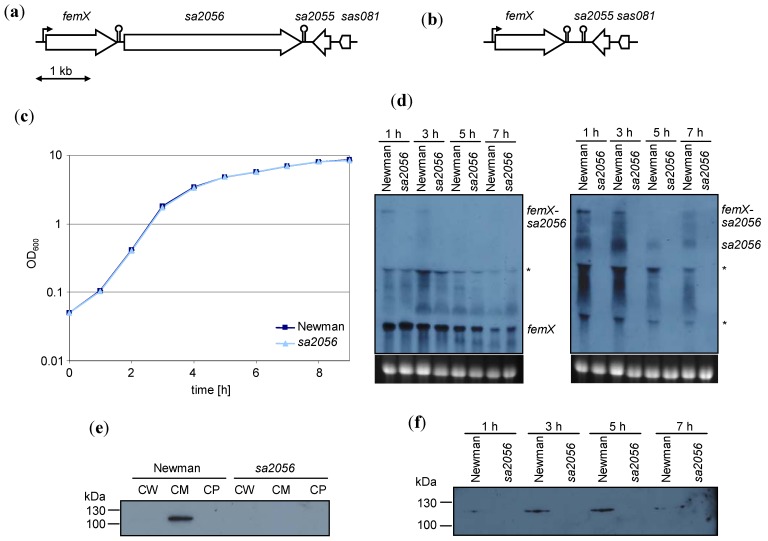
Expression of *sa2056* in strain Newman and its *sa2056* mutant. Genetic organization of the *femX*-*sa2056* region in (**a**) the wild-type and (**b**) the *sa2056* mutant. Construction of the *sa2056* mutant is detailed in supplementary figure S1. (**c**) Growth curves from Luria-Bertani broth (LB) cultures monitored during 9 h. (**d**) Northern blot analyses of RNA samples taken after 1, 3, 5 and 7 h of growth. Digoxigenin (DIG)-labeled probes for *femX* (left panel) and *sa2056* (right panel) were used. Relevant bands are indicated. Ethidium bromide-stained 16S rRNA is shown as an indication of RNA loading. Bands that might be caused by interference of bulk 16S and 23S rRNA are designated by asterisks. Specific antibodies against SA2056 (114.7 kDa) were used for Western blot analyses of (**e**) cell wall (CW), cell membrane (CM) and cytoplasmic (CP) fractions isolated from exponentially growing cells and (**f**) membrane preparations from samples taken after 1, 3, 5 and 7 h of growth.

The transcriptional profile of *sa2056* was determined by Northern blot analyses with specific DIG-labeled probes against *femX* or *sa2056* (Figure 1). *sa2056* was transcribed mainly during exponential growth and partially co-transcribed with *femX*, as a 4.55 kb-transcript could be detected with both probes. Transcriptional start site determination by primer extension did not identify a promoter initiating a *sa2056*-specific mRNA (data not shown), suggesting that the mRNA of approximately 2.9 kb hybridizing only with the *sa2056* probe might result from processing of the 4.55 kb transcript. However, we cannot exclude the presence of an alternative promoter that could be active under different conditions than used here. The hairpin structure between *femX* and *sa2056* might function as a transcriptional or translational attenuator, further regulating SA2056 levels. Of interest, *femX* transcription (1.5 kb) was not altered by the deletion of *sa2056*.

Specific antibodies against a recombinant hexahistidine-tagged SA2056 protein were produced and used for Western blot analyses. SA2056 production was highest in late and post-exponential phase (Figure 1) and could be detected in the membrane part of fractionated wild-type cells and not in the *sa2056* mutant. Thus, *S. aureus* expressed SA2056 during growth, suggesting that it has a function in dividing cells.

### 2.2. Analyses of Mutant Phenotype

Bacteria grew in LB during 7 h or 4 days without any apparent difference between wild-type and mutant concerning optical density or colony forming units as reported before [31]. Increasing or decreasing the temperature to 43 °C or 17 °C and addition of salt (1.5 M NaCl) or sucrose (1 M) to test osmotic stress conditions did not lead to any growth difference that might have indicated altered cell envelope stability. Biofilm formation was determined, but was found to be similar as in the wild-type. Both autolysis and ultrastructure of the cells, as determined by electron microscopy, were unchanged (data not shown).

Resistance levels were tested for different antibiotic classes, including β-lactams, glycopeptides or substances affecting peptidoglycan precursor synthesis and a variety of RND-substrates (Supplementary Table S1). Also, daptomycin was included, because this membrane-active antibiotic had been shown to induce *sa2056* transcription 2.04-fold [17]. However, MICs were virtually identical in wild-type and mutant. The *sa2056* mutant was found to be only moderately more resistant to hypochlorite compared to the parent (growth at 5 mM respectively 2.5 mM hypochlorite). No change in resistance was found for mitomycin, mupirocin, peracetic acid or puromycin (data not shown).

To see whether the presence of the methicillin resistance-mediating PBP, PBP2a, had any influence, we transformed Newman and Newman *sa2056* with the plasmid pME2, encoding *mecA* controlled by its promoter [32]. However, there was no difference in the expression of homogeneous resistance as deduced from population analysis profiles on oxacillin (data not shown).

These data suggested that, although expressed, *sa2056* is not required for *S. aureus* growth or stress tolerance under the conditions tested.

### 2.3. Topology of SA2056

**Figure 2:**
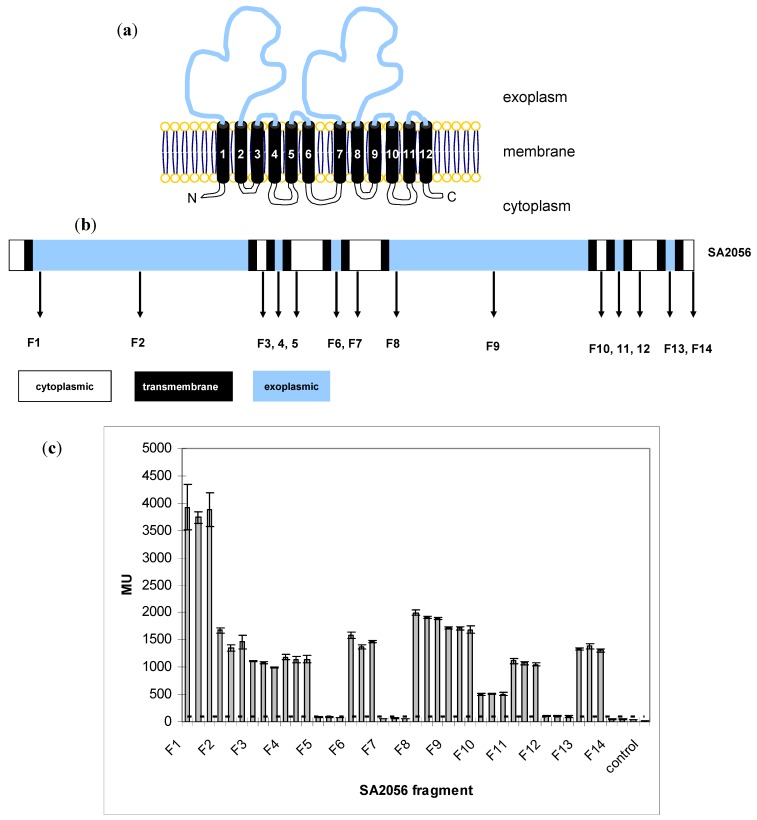
Analysis of SA2056 topology. (**a**) Model of SA2056 topology depicting cytoplasmic (white), membrane (black) and exoplasmic (blue) segments. The N- and C-terminus of the protein are both predicted to be located in the cytoplasm. (**b**) SA2056 fragments F1–14 cloned to the N-terminus of PhoA. (**c**) Activity of fusion proteins was measured in biological and technical triplicates; mean values for each clone are given, and the standard deviation is indicated. SA2056 fragments directing PhoA to the exoplasm were expected to produce values at least five times higher than the background levels (dashed line) measured in the *phoA*-negative *E. coli* strain CC118 (control).

The protein SA2056 is predicted to have 12 transmembrane (TM) domains and two large exoplasmic loops, displaying a typical RND topology with an internal symmetry [11]. Using the TMHMM program, putative TM regions were determined, and fragments containing increasing numbers of TMs (Supplementary Figure S2) were fused to the N-terminus of the *E. coli* alkaline phosphatase PhoA [33]. PhoA is widely used in topology studies, because it folds only in the exoplasm into an enzymatically active conformation [34,35,36]. Expression of the fusion proteins was confirmed by Western blot analyses (data not shown), and PhoA activity was determined (Figure 2). Topology was confirmed, except for fragments ending after TM2 and TM8. Sequence analyses with other membrane prediction programs (DAS, SOSUI, HMMTOP, MEMSAT) revealed ambiguities regarding the end of TM2 and the start of TM3, for which additional F3 constructs were made (F3a–e; S2) by adding up to five amino acids. However, all of the constructs directed PhoA to the exoplasm, suggesting that the short stretch between TM2 and TM3 might not allow PhoA to protrude into the cytoplasm. Similarly, the same might be true for TM8 and TM9, which are separated by 3–6 aa, depending on the program. Taken together, we could confirm the overall topology and show that SA2056 covers cytoplasmic, membrane and exoplasmic spaces. Thus, SA2056 has the potential to interact with proteins present in these different subcellular locations. 

To monitor the localization of SA2056 in the cell, the green fluorescent protein (GFP) was fused to the C-terminus of SA2056, and expression of SA2056-GFP was visualized in exponentially growing cells (Supplementary Figure S3). Quantification of the fluorescence signal of the hemispherical and septal membrane showed that the signal from the septum was approximately twice as high, suggesting that the increased brightness was caused by the presence of two instead of one plasma membrane at the septum and not by a preferred localization of SA2056 to the septum. However, because of the relatively high background signal in the cytoplasm, no ultimate conclusion about the localization of SA2056 could be drawn. Discrete fluorescent patches, which could reflect a heterogeneous distribution of SA2056 in the membrane, were also observed in the membrane in dividing and non-dividing bacteria and were not influenced by the addition of methicillin (data not shown). 

### 2.4. Interactions of SA2056 Identified in a Bacterial Two-Hybrid System

In parallel to the construction of a *sa2056* deletion mutant, interaction studies were performed using a bacterial two-hybrid system (BACTH) developed by Karimova *et al*. [37]. To test whether there was a physical link to peptidoglycan synthesis besides the transcriptional coupling to *femX*, interactions with the FemABX factors and the PBPs were examined. As the *E. coli* RND protein AcrB had been reported to form trimers [38], SA2056 was also tested for interaction with itself. Candidates were fused to the *Bordetella pertussis* adenylate cyclase CyaA domains T18 and T25, as described in Materials and Methods. pKT25 and pUT18-vectors encoding the fusion proteins were co-transformed into the *cya* negative *E. coli* reporter strain DHM1. Co-transformants were plated on MacConkey agar containing lactose as the only carbon source. Interacting partners bring the T18 and T25 domains close enough together to allow them to regain their catalytic function, *i.e.*, the conversion of ATP into cAMP. Production of cAMP was monitored on indicator plates, where the expression of cAMP-dependent enzymes, such as β-galactosidase, leads to the degradation of lactose, acidification of the medium and color formation. To estimate the strength of interaction, β-galactosidase activity was determined. As negative controls, vectors encoding only T18 or T25 were combined with candidate proteins.

**Figure 3:**
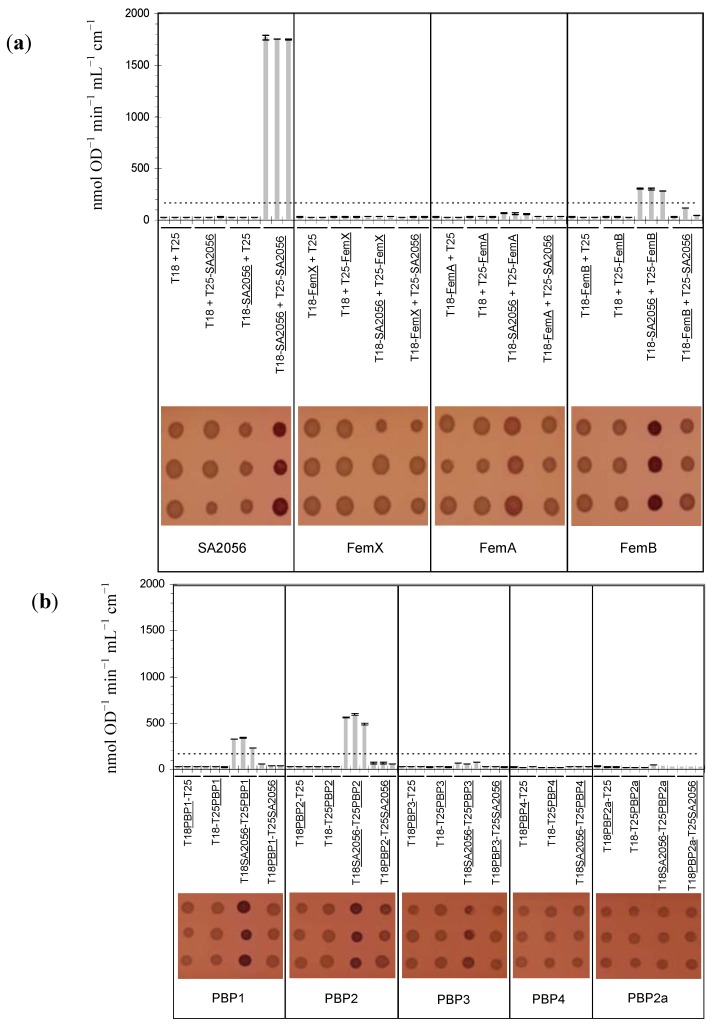
SA2056 interactions determined using the bacterial two-hybrid system. (**a**) SA2056 interactions with itself and the FemABX factors. (**b**) SA2056 interactions with penicillin binding proteins (PBPs). Three representative co-transformants containing the plasmids indicated were analyzed regarding β-galactosidase activity, which was determined by measuring the formation of o-nitrophenol (top). Means of three technical replicates and their standard deviation are shown. The threshold corresponding to the highest negative control value multiplied by five is indicated by a dashed line. Alternatively, the ability of co-transformants to degrade lactose to lactate was tested on MacConkey agar (bottom), where acidification of the medium leads to pink colonies.

For pUT18-*pbp4*/pKT25-*sa2056* co-transformations, no viable clones were obtained; the same plasmids were used successfully in other transformations, suggesting that this particular combination was unfavorable for the cells and that the plasmids themselves were not toxic. The interaction of PBP4 with SA2056 was therefore tested only in one orientation. For every combination, three representative clones were analyzed (Figure 3). The highest value of all negative controls was multiplied by five to set the threshold for significant interactions. SA2056 was found to strongly interact with itself, suggesting that, although not extruding RND substrates, this protein has the potential to form homotrimers like other RND proteins. Possibly due to an unfavorable conformation of the T25-SA2056 protein, SA2056 was able to interact with FemB, PBP1 and PBP2 only when fused to the T18 domain.

**Figure 4:**
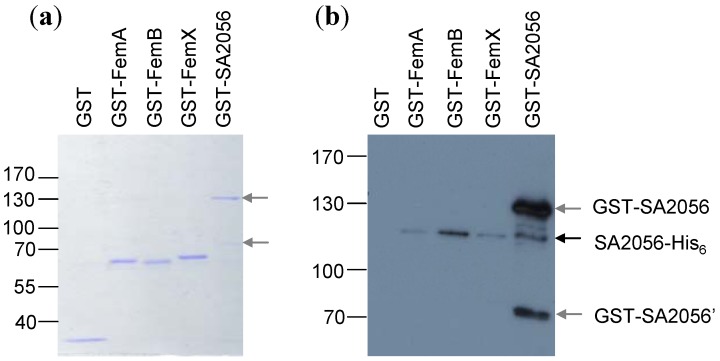
Pull-down experiments. Recombinant glutathion-S-transferase (GST)-tagged SA2056 and FemABX proteins were incubated with SA2056-His_6_ and aliquots of bound proteins were separated on denaturing polyacrylamide gels for Coomassie staining (**a**) or (**b**) transferred to a polyvinylidene fluoride (PVDF)-membrane for Western blot analysis using specific antibodies against SA2056. Relevant bands are indicated. GST (26 kDa), GST-FemA (74.1 kDa), GST-FemB (74 kDa), GST-FemX (74.3 kDa), SA2056-His_6_ (115 kDa). Probably due to degradation, an additional band (~70 kDa; GST-SA2056’) was visible in the preparation of GST-SA2056 (147.2 kDa) and detected with the antibodies. For appropriate separation, a 10%-(a) and a 7.5%-polyacrylamide gel (b) were used. GST-tagged proteins had a tendency to run slightly faster than expected from their predicted mass.

To exclude that endogenous *E. coli* proteins were mediating or hindering interactions, pull-down experiments with purified recombinant proteins were performed. Proteins were tagged with glutathion-S-transferase (GST) or a hexahistidine (His_6_) peptide. SA2056-GST or GST alone was bound to glutathion-sepharose, blocked with BSA and incubated with His_6_-tagged interaction candidates. After washing, bound proteins were detached with sample buffer and separated on denaturing polyacrylamide gels (Figure 4). Similar amounts of GST-tagged bait proteins were present in the reactions, as determined by Coomassie staining (Figure 4a). SA2056 was confirmed to interact with itself and FemB and was found to very weakly interact also with FemA and FemX under these conditions (Figure 4b). Pull-down experiments with recombinant PBP-His_6 _proteins were unsuccessful, possibly requiring further optimization of assay conditions allowing PBPs to interact with SA2056. For instance, co-factors might be required that are only present in the cell or a membrane environment.

### 2.5. Resistance Phenotype of a femB sa2056 Double Mutant

Since FemB was found in both *E. coli* and *in vitro* experiments to interact with SA2056, a *femB sa2056* double mutant was constructed to determine whether the lack of *sa2056* in the compromised *femB* single mutant leads to a phenotype. A *femB* transposon mutation was transduced into the Newman *sa2056* mutant, and the resulting *femB sa2056* double mutant was tested for any alterations in β-lactam or lysostaphin resistance compared to the *femB* mutant, which is known to have a reduced β-lactam and an increased lysostaphin resistance [39,40]. However, the *femB sa2056* mutant showed similar resistance to cefoxitin and lysostaphin, as did the single mutant *femB* (data not shown), suggesting that under the conditions tested *sa2056* does not play a major role in *S. aureus,* and thus, a major function in the last steps of peptidoglycan precursor synthesis is unlikely. This was also supported by testing of recombinant SA2056 in the previously described *in vitro* peptidoglycan synthesis assay, where no alterations in pentaglycine interpeptide synthesis was found upon addition of SA2056 (data not shown) [4].

In *S. aureus*, two additional RND proteins are present: SecDF and SA2339, a homologue of MmpL that may be involved in lipid transport. While the importance of SecDF for *S. aureus* resistance and expression of virulence factors has been recently described, the function of SA2339 has not yet been identified. Like *sa2056*, deletion of *sa2339* leads to no phenotype regarding growth or resistance [31]. It is possible that SA2056 and SA2339 share functional redundancy, similar to the *S. aureus* LytR-CpsA-Psr proteins [41] and that deletion of just one of the genes does not impair *S. aureus* sufficiently to produce a phenotype. Alternatively, SA2056 might be part of a complex network involving several players, where the absence of just one factor has little impact on *S. aureus* under the conditions tested here. 

## 3. Experimental

### 3.1. Bacterial Strains and Growth Conditions

Strains and plasmids used in this study are listed in Supplementary Table S2. Bacteria were grown aerobically at 37 °C in Luria-Bertani broth (LB), where nothing else is mentioned. Good aeration for liquid cultures was assured by vigorously shaking flasks with an air-to-liquid ratio of at least 4. For growth curves, strains were grown in triplicate, and means with standard deviations were determined.

Antibiotic susceptibilities were determined using Etest strips (AB-Biodisk, Solna, Sweden), containing exponential gradients of active components, on MH agar plates with an inoculum of a 0.5 McFarland standard, corresponding to 10^8^ cells/mL. Minimal inhibitory concentrations (MICs) were read after 24 h of incubation. Alternatively, broth microdilution methodology was used. For qualitative susceptibility determination, bacterial 0.5 McFarland suspensions were swabbed across agar plates containing appropriate concentration gradients of test substances.

To sample RNA and protein, cells from overnight cultures were used to inoculate prewarmed LB to an optical density at 600 nm (OD_600_) of 0.05, corresponding to 10^7^ cells/mL.

### 3.2. Construction of MRSA Strains

The plasmid pME2 containing the *mecA* promoter and gene from strain COLn [32] was introduced into strains of interest, as described in [31].

### 3.3. Northern Blot Analyses

Total RNA was isolated as described previously [42] by using a FastRNA kit and a Fastprep reciprocating shaker (Bio 101). For Northern blots, 5 to 10 μg of total RNA per lane was separated on a 1.5% agarose-20 mM guanidine thiocyanate gel and transferred overnight onto a positively charged nylon membrane (Roche, Rotkreuz, Switzerland). The blots were hybridized with specific digoxigenin-labeled DNA probes, which were produced using a PCR DIG probe synthesis kit (Roche). Primers used are listed in Supplementary Table S3. Data shown were confirmed in at least two independent experiments.

### 3.4. Expression of Recombinant Proteins

Genes encoding FemABX, SA2056, PBP1-4 and 2a were amplified from genomic DNA using primers listed in supplementary table T3. Amplicons were cloned into pET24b(+) using NheI/XhoI or BamHI/XhoI. In the case of pGEX-2T, PCR products were inserted into BamHI/EcoRI digested plasmids.

His_6_-tagged and GST-tagged FemABX factors were purified as described in [43]. For the membrane proteins SA2056 and the PBPs, the *E*. *coli* BL21 derivative CE43 was used, since it had been selected for increased membrane protein production [44]. Transformants were grown in LB at 37 °C. At an OD_600_ of 1.5, the expression of the recombinant proteins was induced with 1 mM isopropyl-β-D-thiogalactopyranoside (IPTG). Bacteria were then grown for 20 h at 25 °C and were collected by centrifugation.

For purification of His_6_-tagged proteins, pellets were resuspended in 50 mM Tris-HCl pH 7.5, 500 mM NaCl, 20 mM imidazol and 0.5% N-lauroylsarcosine. Protease inhibitors (Complete EDTA-free, Roche) were added as recommended by the manufacturer. Cells were lysed on ice for 30 min with 2 mg/mL lysozyme, 0.15 mg/mL DNase and 0.075 mg/mL RNase. The cleared lysate was gently mixed for 2 h at 4 °C with Ni-NTA beads (Qiagen, Hombrechtikon, Switzerland). Beads were collected and washed with 50 mM Tris/HCl pH 7.5, 500 mM NaCl, 20 mM imidazol, followed by a second wash with the same buffer containing 50 mM imidazol. Proteins were eluted with 50 mM Tris/HCl pH 7.5, 500 mM NaCl, 1 mM *n*-dodecyl-β-D-maltoside (DDM), 100 mM imidazol followed by a second round of elution with the same buffer containing 200 mM imidazol. Proteins were stored at 4 °C or, supplied with 20% glycerol, at −20 °C.

GST-tagged proteins were isolated similarly with minor changes: Resuspension was done in 50 mM Na_2_HPO_4_ pH 7.8, 150 mM NaCl, 10 mM DDM. Glutathion (GSH)-Sepharose 4B (GE Healthcare) was used for binding of the GST-tagged proteins, which were washed once with the same buffer and then eluted with 10 mM GSH, 50 mM Tris-HCl pH 8. Ten mM DDM was added only in the case of the membrane proteins SA2056, PBP1-4 and 2a.

### 3.5. S. aureus Cell Fractionation

Based on the method used by Schneewind *et al.* [45], cell wall, cell membrane and cytoplasmic fractions of bacteria grown until an OD_600_ of 1 were prepared, as described in [31]. Representative data from two independent experiments are shown.

### 3.6. Western Blot Analyses

Recombinant His-tagged SA2056 was prepared as described above and used for the production of specific rabbit antibodies (Davids Biotechnology, Regensburg, Germany). For Western blots, 10 μg protein was loaded and separated by SDS-10% polyacrylamide gel electrophoresis. Page Ruler (Thermo Scientific, Waltham, MA, USA) was used as a molecular size marker. Gels were either stained with Coomassie or transferred onto nitrocellulose (Hybond; Amersham Biosciences, Glattbrugg, Switzerland) or polyvinylidene fluoride (PVDF; Immobilon-P, Millipore, Zug, Switzerland) membranes. Membranes were blocked with skim milk and preincubated with 40 μg/mL human immunoglobulin G (Calbiochem, Darmstadt, Germany) to saturate any immunoglobulin-binding proteins and, thereby, prevent cross-reactivity of antigen-purified rabbit antibodies against SA2056. Horseradish peroxidase-conjugated goat anti-rabbit secondary antibody (Jackson ImmunoResearch Laboratories, Inc., Suffolk, UK) was diluted 1:10,000 and detected with SuperSignal West Pico solutions (Thermo Scientific). 

### 3.7. Construction of *phoA* Fusions and PhoA Activity Assay

The gene fragments of interest were cloned into the 5' XhoI and 3' KpnI sites of plasmid pHA-1, which carries a *phoA* gene lacking both the 5' segment coding for the signal sequence and the first five residues of the mature protein [36]. Between the SA2056-fragment and the PhoA moiety, an 18 amino acid linker was present. The constructs were transformed into the *phoA*-negative *E. coli* strain CC118, which is not able to use arabinose and galactose. For each construct, three transformants were used for the determination of PhoA activity.

Three mL LB medium was inoculated with 30 μL overnight culture and grown at 37 °C to an OD_600_ of 0.5. The protein expression was induced with 0.2% arabinose during 1 hour at 37 °C. One mL of the culture was centrifuged at maximal speed in a microcentrifuge, washed with 1 mL ice cold 1 M Tris-HCl (pH 8.0) and resuspended in 1 mL ice cold 1 M Tris-HCl (pH 8.0). After measuring the OD_600_, 3 aliquots (0.05, 0.1, 0.2 mL) were adjusted to a total volume of 0.5 mL with ice cold 1 M Tris-HCl (pH 8.0). Cells were permeabilized by adding 20 μL chloroform and 10 μL 0.01% SDS and incubation at 37 °C for 5 min. Addition of 0.5 mL of 2 mM p-nitrophenyl phosphate (pNPP, Sigma-Aldrich, St. Louis, MO, USA) in 1 M Tris-HCl (pH 8.0) initiated the reaction. After 10 min incubation at 37 °C, the reaction was stopped by adding 0.2 mL 0.5 M K_2_HPO_4_ (pH 8.0). The cell debris was removed by centrifugation, and the absorption was measured at 550 and 420 nm to determine residual cell debris, respectively, and color formation by pNPP degradation. Miller Units were calculated using the following formula:

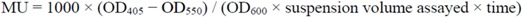



### 3.8. Topology Prediction Programs

Five topology prediction programs were used: TMHMM [46], DAS [47], HMMTOP [48], MEMSAT [49], SOSUI [50]. 

### 3.9. Bacterial Two-Hybrid System

Candidate genes were amplified from genomic DNA using primers listed in Supplementary Table S3 and cloned into pUT18 and pKT25 vectors [37] using the restriction sites PstI and KpnI. Fifty μL of RbCl-competent DHM1 cells were co-transformed with 20 ng of each plasmid and plated on Difco MacConkey agar containing lactose (BD, No. 212123), 25 μg/mL kanamycin (Km) and 100 μg/mL ampicillin (Ap). Transformants were grown at 30 °C, and three representative clones from a minimum of 50 clones were restreaked for further experiments. One mL LB with 0.5 mM IPTG, 25 μg/mL Km, 100 μg/mL Ap was inoculated with one clone and grown at 30 °C for 16 h. Cells were centrifuged and stored at −20 °C until use. For spotting, 0.5 mL LB was inoculated with 5 μL overnight culture, of which 0.1 mL were transferred into microtiter dishes and used for spotting on MacConkey agar plates. For determination of β-galactosidase activity, frozen pellets were resuspended in 1 mL Z buffer (60 mM Na_2_HPO_4_, 40 mM NaH_2_PO_4_, 10 mM KCl, 1 mM MgSO_4_ pH 7) and OD_600_ was determined. 1 mL Z buffer was used as control and treated like the samples. For each clone, measurements were made in triplicate. One hundred μL aliquots were added to 900 μL Z buffer, 35 μL chloroform, and 35 μL 0.1% SDS was added. Cells were vortexed during 10 s for permeabilization, followed by an incubation step at 28 °C for 5 min. 0.2 mL of a 0.4% *o*-nitrophenol-β-galactopyranoside (ONPG) solution in buffer Z was added and incubated for 5 min at 28 °C. The reaction was stopped by adding 0.5 mL of a 1 M Na_2_CO_3_ solution. Cell debris was removed by centrifugation during 1 min at full speed. The supernatant was used to measure the absorption of the β-galactosidase product *o*-nitrophenol at 420 nm and to determine light scattering of any remaining particle at 550 nm. Activity, one unit corresponding to the hydrolyzation of 1 nmol of ONPG per min at 28 °C, was calculated using the following formula and is given in nmol OD^−1^ min^−1^ cm^−1^:

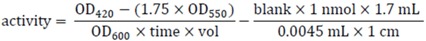



### 3.10. Pull-Down Experiments

Per reaction, 2 μg recombinant GST-tagged protein was bound to 10 μL of GSH-sepharose 4B slurry by mixing 1 h at 4 °C in binding buffer (50 mM Na_2_HPO_4_ pH 7.8, 150 mM NaCl, 1 mM DDM). Sepharose was washed four times with binding buffer and blocked with 5 mg/mL bovine serum albumin (BSA) in binding buffer for 0.5 h at 4 °C. Sepharose was washed four times with interaction buffer (40 mM Tris-HCl pH 7.5, 10 mM MgCl_2_, 1 mM DDM, 0.2% BSA). Two μg recombinant His_6_-tagged protein was added, and the reactions were mixed for 1 h at room temperature. Sepharose was washed four times with wash buffer (40 mM Tris-HCl pH 7.5, 10 mM MgCl_2_, 1 mM DDM, 50 mM NaCl). Bound material was detached by adding 20 μL of sample buffer (4.4 M urea, 2.7% non-idet P-40, 2.7% β-mercaptoethanol, 0.16 M Tris-HCl pH 6.8, 6.2% SDS, 4.5% glycerol, bromophenol blue) and heated for 5 min at 65 °C. 7 μL were separated on a denaturing 7.5 or 10% polyacrylamide gel. 10%-gels were stained with Coomassie to visualize GST-tagged proteins and to exclude that any other *E. coli* proteins were present that might have mediated interactions. 7.5%-gels were blotted onto a PVDF-membrane and used for Western blots with specific rabbit anti-SA2056 or rabbit anti-His_6_ (Abcam) antibodies, which were detected as described above. Representative data from two independent experiments are shown.

## 4. Conclusions

Under standard laboratory conditions, the yet uncharacterized RND protein, SA2056, is expressed in *S. aureus* and, thus, must be assumed to have a function. SA2056 can interact with itself, suggesting that it could form a trimer and work as an efflux pump; the substrate is likely to be very specific, as deletion of *sa2056* had no influence on resistance against typical RND substrates or a range of antibiotics. The interaction found between SA2056 and FemB using two different methods hints at a possible, subsidiary role in peptidoglycan synthesis or cell division, but could also be of a more general nature in coordinating processes occurring at the membrane. This yet uncharacterized role could represent a novel aspect of the functional diversity of RND proteins. In the case of *S. aureus*, the function of SA2056 might be redundant with a second uncharacterized RND protein, SA2339, or be of importance only under certain conditions that were not tested here and remain to be identified. 

## Supplementary Files

Supplementary File 1
