# Supplementary material for: The Staphylococcus aureus Membrane Protein SA2056 Interacts with Peptidoglycan Synthesis Enzymes
Source: Antibiotics (Basel). 2013 Jan 22;2(1):11–27. doi: 10.3390/antibiotics2010011 (PMC4790295; doi:10.3390/antibiotics2010011)
Supplement: Supplementary File 1 [file antibiotics-02-00011-s001.pdf]

Supplementary Material

Supplementary Figure S1. Construction of the *sa2056* mutant [1]. *sa2056* was excised by a three-step procedure developed by Bae *et al.* [2]: First, the temperature-sensitive plasmid pCQ30 was integrated at 43 °C either up- or down-stream of *sa2056* by homologous recombination. Only the resulting chromosomal organization of the recombination symbolized on the left is given. Next, the plasmid was allowed to excise together with the *sa2056* gene at permissive temperature (30 °C). Finally, bacteria were selected for plasmid loss.

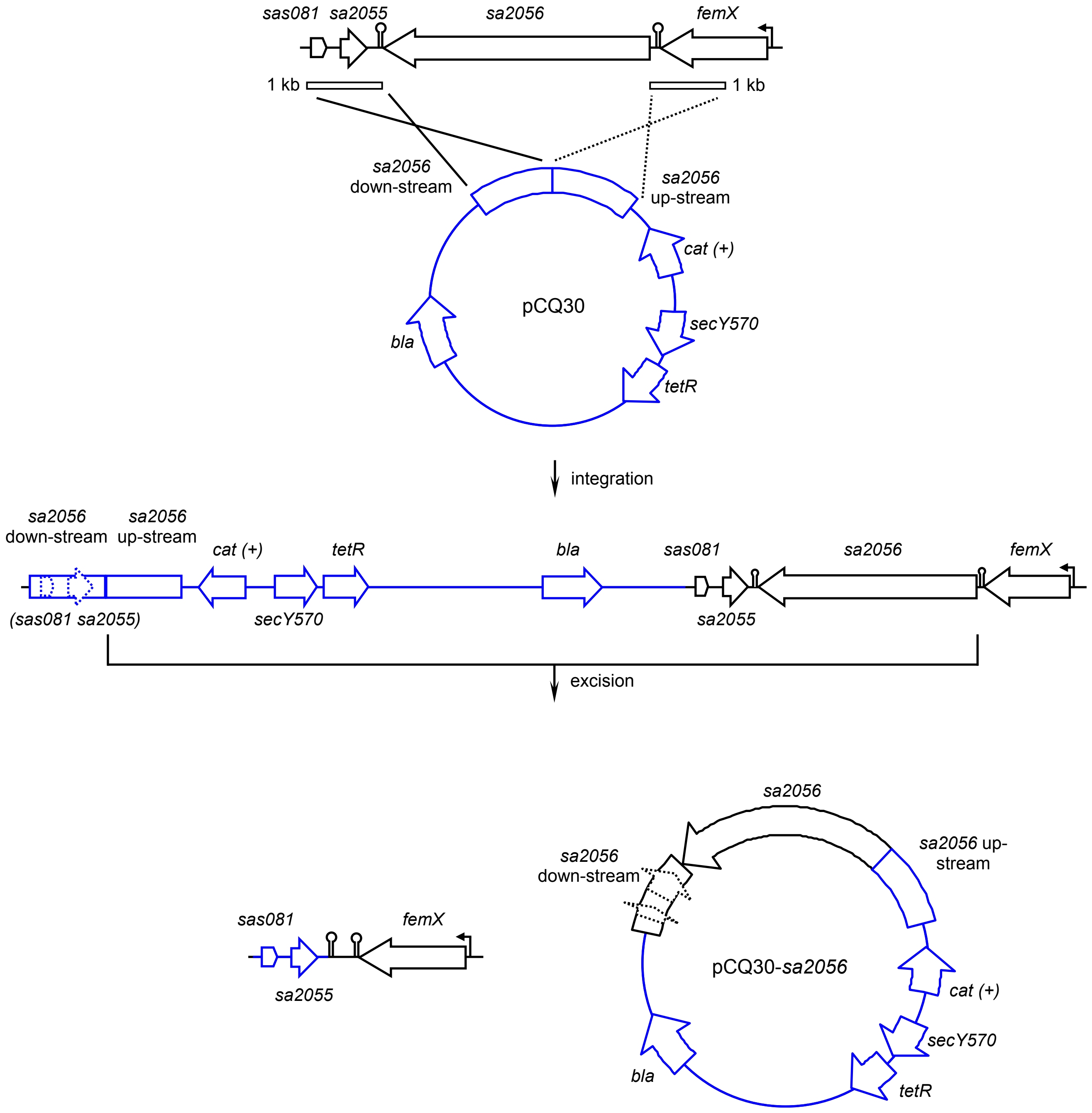

Supplementary Figure S2. (**a**) Amino acid sequence of SA2056. Transmembrane (TM) regions predicted by THMMH and C-termini of fragments (F) fused to PhoA are indicated. For TM2 and TM3, predictions of additional programs are depicted. Extra amino acids added to F3 are indicated (F3a–e). (**b**) Activity of fusion proteins was measured in biological and technical triplicates; mean values for each clone are given and the standard deviation is indicated. SA2056 fragments directing PhoA to the exoplasm were expected to produce values at least five times higher than the background levels (dashed line) measured in the *phoA*-negative *E. coli* strain CC118 (control).

(a)

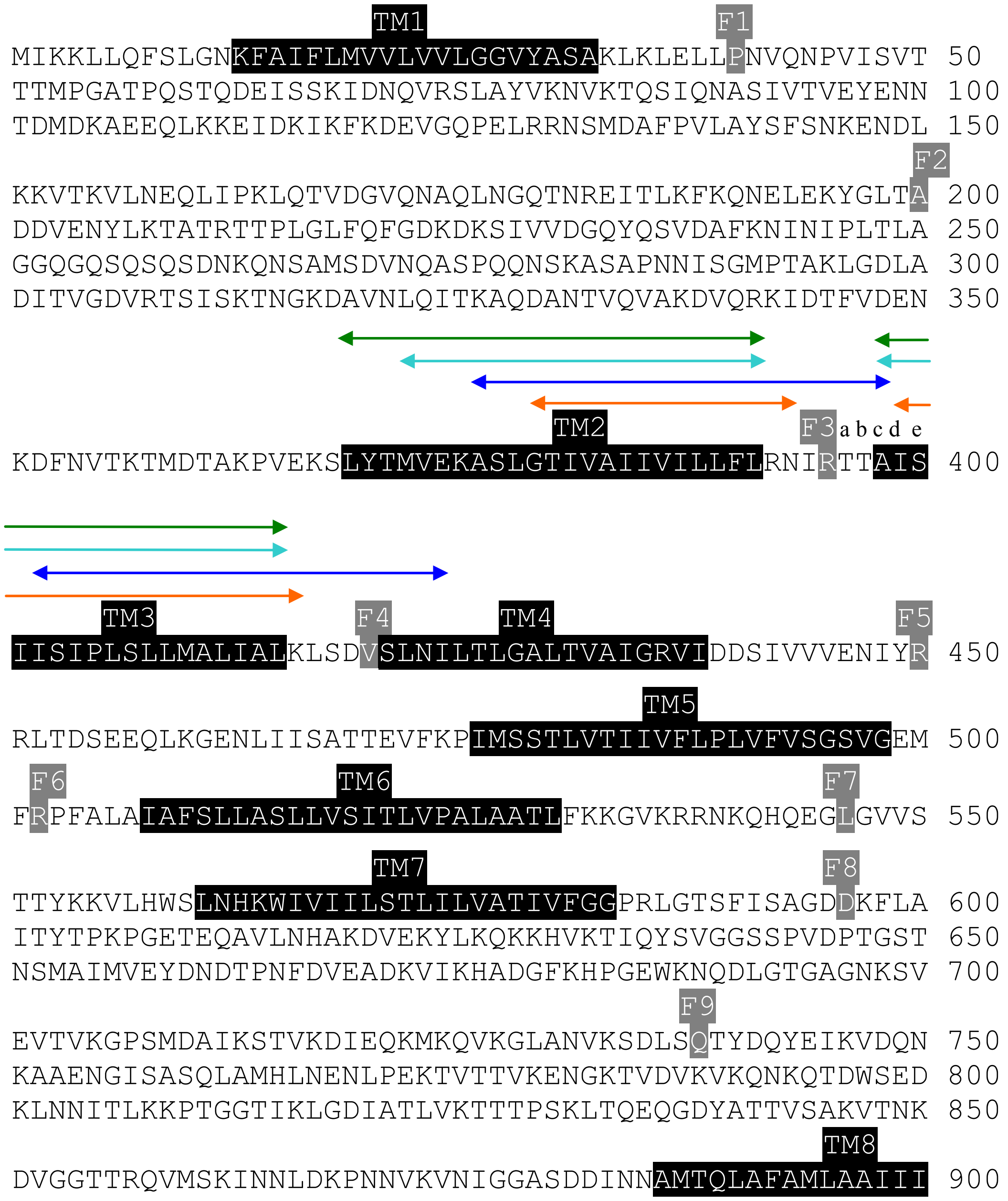

F10 TM9 F11
VYLILVITFKGGLAPFTILFSLPFTVIGVIIALLITGETISVPSLIGMLM 950

TM10 F12 TM11
LIGIVVTNAIVLIDRVINNEQQGMEMKEALIEAGGTRIRPILMTAIATIG 1000

F13 TM12
ALVPLLFGQDSSILISKGLAATVIGGLISSTLLTLVVVPVIYEILFTLKK 1050

F14
RFTKR

	TMHMM	DAS	SOSUI	HMMTOP	MEMSAT
**TM2**	369-391	379-392	376-398	372-391	375-391
**TM3**	398-415	399-416	402-424	398-415	398-415

(b)

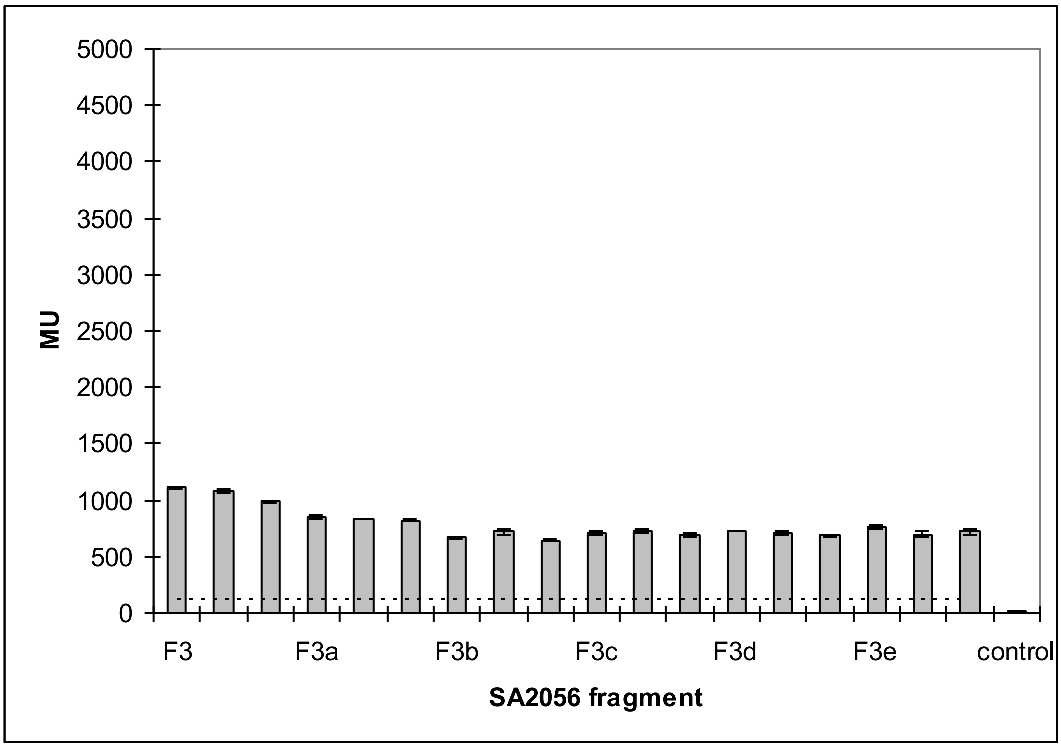

Supplementary Figure S3. Localisation of SA2056. Exponentially grown *S. aureus* expressing SA2056-GFP under the control of the *sa2056* promoter was visualised by (**a**) phase contrast or (**b**) fluorescence microscopy as described below. Arrows indicate examples of dividing bacteria with visible septa and SA2056-patches. Bars indicate the size of 1 μm.

(**a**)

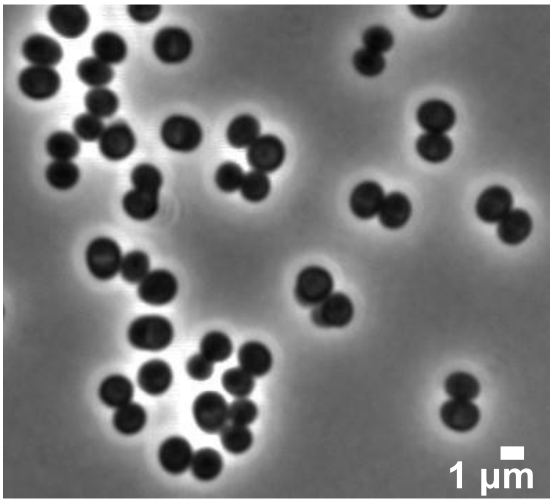

(**b**)

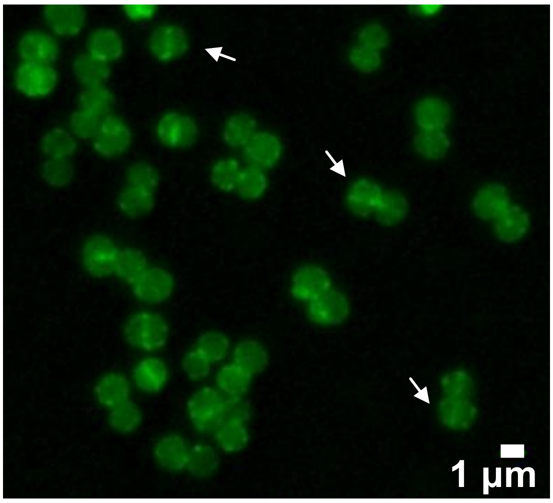

The 3'-region of SA2056 ($SA2056_{666nt}$) was amplified from genomic DNA using primers listed in supplementary table T2. The $SA2056_{666nt}$ fragment was cloned to the 5' end of *gfpmut1* in pSG5082 using the XhoI and HindIII restriction sites, yielding pCQ44 [3]. Following the transformation of pCQ44 into *E. coli* DH5α (CQ44), the suicide vector was integrated into *S. aureus* RN4220 (CQ48). To confirm correct integration, a PCR with subsequent sequencing of the region was performed.

CQ48 was grown in tryptic soy broth (TSB, Difco) until exponential phase, washed once in PBS (8 g NaCl, 0.2 g KCl, 2.68 g Na_2HPO_4-$7H_2O$, 0.24 g KH_2PO_4, pH 7.4) and resuspended therein. A drop of bacterial suspension was spotted on a microscope slide overlaid with a thin layer of 1 % agarose in PBS and covered with a cover slip. Cells were visualised using a Zeiss Axio Observer.Z1 microscope and the Metamorph v. 7.5 software (Molecular Devices). Pictures were acquired with the Photometrics CoolSNAP HQ2 camera (Roper Scientific), which was connected to the microscope. Pictures were analysed with the ImageJ software [4].

Supplementary Table S1. Resistance profiles of strains Newman and *sa2056*.

		MIC [µg/mL]	
	**Substance**	**Newman**	***sa2056***
Cell wall synthesis inhibitors	Cefoxitin	6	6
	Oxacillin	0.38	0.38
	Teicoplanin	4	6
	Vancomycin	5	6
	Lysostaphin	0.125–0.25	0.125–0.25
	D-cylcoserine	8	8
	Fosfomycin	0.25	0.25
	Ramoplanin	1	1
	Nisin	4	4
	Mersacidin	32	32
	Bacitracin	8	8
RND substrates	Acriflavine	8	8
	EtBr	1–2	1–2
	SDS	64	64
Others	Daptomycin	2	2
	Clindamycin	0.94	0.94
	Chloramphenicol	4	3
	Tetracycline	0.19	0.25
	Gentamicin	0.75	1
	Erythromycin	0.25	0.25
	Novobiocin	0.0313	0.0313
Fatty acids	Capric acid	512	512
	Linoleic acid	16	16
	Cis-6-hexadecenoic acid	64	64

Supplementary Table S2. Strains and plasmids used in this study.

Strains	Relevant genotype and phenotype	Reference or source
S. aureus		
Newman	Clinical isolate (ATCC 25904), *rsbU*$^{+}$	[5]
RN4220	NCTC 8325-4 r^{-} m^{+}	[6]
CQ33	Newman Δ*sa2056*	[1]
CQ38	Newman Δ*sa2056* pME2, Tc^{r}, Mc^{r}	[1]
CQ39	Newman pME2, Tc^{r}, Mc^{r}	[1]
CQ48	RN4220 *sa2056*::pCQ44, SA2056-GFP, Em^{r}	This study
MS146	Newman *femB*::Tn551, Em^{r}, Lss^{r}	This study
MS147	Newman Δ*sa2056 femB*::Tn551, Em^{r}, Lss^{r}	This study
UT34-2	NCTC 8325 *mec* Ω2006(*femB*::Tn551), Em^{r}, Lss^{r}	[7]
E. coli		
BL21	Expression strain, DE3 (*E. coli* B F^{-} *ompT hsdS*$_{B}$ *gal dcm*),λ prophage carrying T7 polymerase	Novagen
CE43	Membrane protein overproducer selected from BL21	[8]
CC118	Reporter strain for PhoA fusion, Δ(*ara-leu*)7697 Δ*lacX74* Δ*phoA20 galE galK*	[9]
CQ44	DH5α pCQ44, Ap^{r}	This study
DH5α	Cloning strain (F− Φ80*lac*ZΔM15 Δ(*lac*ZYA-*arg*F)U169 *rec*A1 *end*A1 *hsd*R17 (rk−, mk+) *pho*A *sup*E44 *thi*-1 *gyr*A96 *rel*A1 λ−]	Invitrogen
DHM1	BACTH reporter strain, *cya*	[10]
**Plasmids**		
pCQ44	Suicide vector, SA2056$_{666nt}$-GFP fusion at C-terminus, Ap^{r}, Em^{r}	This study
pET24b(+)	Expression vector, N-terminal T7-Tag or C-terminal His$_{6}$-Tag, T7 promoter, Km^{r}	Novagen
pET24b(+)-*femA*	Expression vector, *femA* with His$_{6}$-Tag at the C-terminus, Km^{r}	[11]
pET24b(+)-*femB*	Expression vector, *femB* with His$_{6}$-Tag at the C-terminus, Km^{r}	[11]
pET24b(+)-*femX*	Expression vector, *femX* with His$_{6}$-Tag at the C-terminus, Km^{r}	This study
pET24b(+)-*sa2056*	Expression vector, *sa2056* with His$_{6}$-Tag at the C-terminus, Km^{r}	This study
pGEX-2T	Expression vector, N-terminal GST-Tag, Ap^{r}	GE Healthcare
pGEX-2T-*femA*	Expression vector, *femA* with GST-Tag at the N-terminus, Ap^{r}	This study
pGEX-2T-*femB*	Expression vector, *femB* with GST-Tag at the N-terminus, Ap^{r}	This study
pGEX-2T-*femX*	Expression vector, *femX* with GST-Tag at the N-terminus, Ap^{r}	This study
pGEX-2T-*sa2056*	Expression vector, *sa2056* with GST-Tag at the N-terminus, Ap^{r}	This study
pHA-1(*yedZ*)	PhoA fusion expression plasmid containing *yedZ* (XhoI-KpnI) with *phoA* fused to the 3'-end, *araB* promoter	[12]
pHA-F1-F14	PhoA fusion vectors, *sa2056* fragments encoding F1-F14 fused to the 5'-end of *phoA*	This study
pKT25	BACTH vector, MCS at the C-terminus of the CyaA domain T25, Km^{r}	[10]
pKNT	BACTH vector, MCS at the N-terminus of the CyaA domain T25, Km^{r}	[13]
pKT25-*femA*	BACTH vector, *femA* fused to the C-terminus of T25, Km^{r}	[11]
pKT25-*femB*	BACTH vector, *femB* fused to the C-terminus of T25, Km^{r}	[11]

Table S2. *Cont.*

Strains	Relevant genotype and phenotype	Reference or source
pKT25-*femX*	BACTH vector, *femX* fused to the C-terminus of T25, Km^r	This study
pKT25-*pbp1*	BACTH vector, *pbp1* fused to the C-terminus of T25, Km^r	[14]
pKT25-*pbp2*	BACTH vector, *pbp2* fused to the C-terminus of T25, Km^r	[14]
pKT25-*pbp3*	BACTH vector, *pbp3* fused to the C-terminus of T25, Km^r	This study
pKNT25-*pbp4*	BACTH vector, *pbp4* fused to the N-terminus of T25, Km^r	[14]
pKT25-*pbp2a*	BACTH vector, *pbp2a* fused to the C-terminus of T25, Km^r	This study
pKT25-*sa2056*	BACTH vector, *sa2056* fused to the C-terminus of T25, Km^r	This study
pSG5082	Suicide vector, for c-terminal GFP fusion, Ap^r, Em^r	[3]
pUT18	BACTH vector, MCS at the N-terminus of the CyaA domain T18, Ap^r	[10]
pUT18C	BACTH vector, MCS at the C-terminus of the CyaA domain T18, Ap^r	[10]
pUT18C-*femA*	BACTH vector, *femA* fused to the C-terminus of T18, Ap^r	[11]
pUT18C-*femB*	BACTH vector, *femB* fused to the C-terminus of T18, Ap^r	This study
pUT18C-*femX*	BACTH vector, *femX* fused to the C-terminus of T18, Ap^r	This study
pUT18C-*pbp1*	BACTH vector, *pbp1* fused to the C-terminus of T18, Ap^r	[14]
pUT18C-*pbp2*	BACTH vector, *pbp2* fused to the C-terminus of T18, Ap^r	This study
pUT18C-*pbp3*	BACTH vector, *pbp3* fused to the C-terminus of T18, Ap^r	[14]
pUT18-*pbp4*	BACTH vector, *pbp4* fused to the N-terminus of T18, Ap^r	[14]
pUT18C-*pbp2a*	BACTH vector, *pbp2a* fused to the C-terminus of T18, Ap^r	This study
pUT18C-*sa2056*	BACTH vector, *sa2056* fused to the C-terminus of T18, Ap^r	This study

MCS, multiple cloning site; Ap^r, ampicillin resistant; Cm^r, chloramphenicol resistant; Em^r, erythromycin resistant; Lss^r, lysostaphin resistant; Mc^r, methicillin resistant; Tc^r, tetracycline resistant.

Supplementary Table S3. Primers used in this study.

Primer	Sequence 5'-3'	Use	Reference
CQ10	TCACCCTCTCCACTGACAGA	Confirmation pCQ44 integration	This study
CQ31	AGTGTGGGGAACATACTTAAGTG	Confirmation pCQ44 integration	[3]
CQ33	ATCGAAGCAGGCGGTACTA	Sequencing CQ48	This study
CQ72	TATAAGCTTTCGTTTAGTGAATCGTTT	Construction of pCQ44	This study
CQ73	AAACTCGAGCAAGAACAAGGAGATTATGC	Construction of pCQ44	This study
CQ74	CATCACTTGTCGTGTTGTGC	Sequencing CQ48	This study
EH4	CTGGTACCTTAGTTGAATATACCTGTTAATCCAC	Construction of pUT18C-*pbp2*	This study
EH34	CCGCTCGAGATGATAAAAAAGCTATTAC	Construction of pHA1-F1-14	This study
EH35	CCGGGTACCGGTAGTAATTCTAATTTCA	Construction of pHA1-F1	This study
EH36	ATAGGTACCGCAGTCAACCCATATTTTTC	Construction of pHA1-F2	This study
EH37	ACGGGTACCCGAATGTTTCTTAAAAACAG	Construction of pHA1-F3	This study
EH38	GATGGTACCACATCACTCAATTTCAGAG	Construction of pHA1-F4	This study
EH39	CCGGGTACCCGATAAATATTTTCAACAA	Construction of pHA1-F5	This study
EH40	TATGGTACCCTAAACATTTCGCCTACTG	Construction of pHA1-F6	This study
EH41	ATCGGTACCAATCCTTCTTGATGTTGT	Construction of pHA1-F7	This study
EH42	ATTGGTACCTTATCGTCACCTGCTG	Construction of pHA1-F8	This study
EH43	TATGGTACCTGCGATAAATCAGATTTGAC	Construction of pHA1-F9	This study
EH44	GCGGGTACCCCACCTTTAAATGTAATA	Construction of pHA1-F10	This study
EH45	TAGGGTACCGTTTCTCCTGTGATTAATAG	Construction of pHA1-F11	This study
EH46	TATGGTACCTTCATCTCCATGCCC	Construction of pHA1-F12	This study
EH47	GTGGGTACCGAAATAAGAATCGAGCTAT	Construction of pHA1-F13	This study
EH48	CATGGTACCCGTTTAGTGAATCGTT	Construction of pHA1-F14	This study
EH50	AACTGCAGGACGGAAAACAAAGGATCTTC	Construction of pUT18C-*pbp2*	This study
MS79	ATGGGATCCTGCGAAGCAAAAAATTAAAATTAA	Construction of pET24b-*pbp1*	This study
MS80	TTACTCGAGGTCCGACTTATCCTTG	Construction of pET24b-*pbp1*	This study
MS81	TTGGGATCCCTTAAAAAGACTAAAAGAAAAATCA AATG	Construction of pET24b-*pbp3*	This study
MS82	TTACTCGAGTTTGTCTTTGTCTTTATTTTTATC	Construction of pET24b-*pbp3*	This study
MS83	ATGGGATCCCAAAAATTTAATATCTATTATCATCA TTT	Construction of pET24b-*pbp4*	This study
MS84	TTACTCGAGTTTTCTTTTTCTAAATAAACGATTG	Construction of pET24b-*pbp4*	This study
MS85	ATGGGATCCCAAAAAGATAAAAATTGTTCCACT	Construction of pET24b-*mecA*	This study
MS86	TTACTCGAGTTCATCTATATCGTATTTTTTATT	Construction of pET24b-*mecA*	This study
MS106	CTAGAATTCTTTTCGTTTTAATTTACGAGATATTT	Construction of pGEX-2T-*sa2056*	This study
MS107	CGTGGATCCATAAAAAAGCTATTACAATTTTC	Construction of pGEX-2T-*sa2056*	This study
MS108	CTAGAATTCCTATCGTTTAGTGAATCGT	Construction of pGEX-2T-*sa2056*	This study
MS109	GTAAGATCTGAAAAGATGCATATCACTAATC	Construction of pGEX-2T-*femX*	This study
MS116	GCAGGTACCCTATTTCTTTAATTTTTTACG	Construction of pKT25-*femB* and pUT18C-*femB*	This study
MS117	GTTGAATTCCTATTTCTTTAATTTTTTACG	Construction of pGEX-2T-*femB*	This study
MS118	CTAGAATTCCTATTTTCGTTTTAATTTACGAG	Construction of pGEX-2T-*femX*	This study
MS155	ATTGGTACCGTACGAATGTTTCTTAAAAACAG	Construction of pHA1-F3a	This study
MS156	GAAGGTACCGTCGTACGAATGTTTCTTAAAAAC	Construction of pHA1-F3b	This study
MS157	ATAGGTACCGCCGTCGTACGAATGTTTC	Construction of pHA1-F3c	This study
MS158	GATGGTACCCCAATTGCCGTCGTACGAATGTTTC	Construction of pHA1-F3d	This study

Table S3. *Cont.*

Primer	Sequence 5'-3'	Use	Reference
MS159	GATGGTACCGAAATTGCCGTCGTACGAATG	Construction of pHA1-F3e	This study
SR2	CGAGCTAGCGAAAAGATGCATATCACTAATC	Construction of pET24b-*femX*	[15]
SR3	GCACTCGAGTTTTCGTTTTAATTTACG	Construction of pET24b-*femX*	[15]
SR71	CGTCTCGAGTCGTTTAGTGAATCGTTTTTT	Construction of pET24b-*sa2056*	This study
SR73	GCAGCTAGCATAAAAAAGCTATTACAATTTTCTTT	Construction of pET24b-*sa2056*	This study
SR100	GCACTGCAGGAAATTTACAGAGTTAACTG	Construction of pUT18C-*femB*	[11]
SR101	GCACTGCAGTGAAATTTACAGAGTTAACTG	Construction of pKT25-*femB*	[11]
SR103	CTACTGCAGGGAAAAGATGCATATCAC	Construction of pKT25-*femX*	[11]
SR104	CATCTGCAGTGGAAAAGATGCATATCAC	Construction of pUT18C-*femX*	[11]
SR105	GCAGGTACCTATTTCGTTTTAATTTACG	Construction of pKT25-*femX*	[11]
SR106	GTTGGATCCAAGTTTACAAATTTAACAGCTA	Construction of pGEX-2T-*femA*	[11]
SR107	GTTGAATTCCTAAAAAATTCTGTCTTTAACTTT	Construction of pGEX-2T-*femA*	[11]
SR108	CAAGGATCCAAATTTACAGAGTTAACTGTTAC	Construction of pGEX-2T-*femB*	[11]

Restriction sites are underlined.

References and Notes

1. Quiblier, C.; Zinkernagel, A.; Schuepbach, R.; Berger-Bächi, B.; Senn, M. Contribution of SecDF to *Staphylococcus aureus* resistance and expression of virulence factors. *BMC Microbiol.* **2011**, *11*, doi:10.1186/1471-2180-11-72.
2. Bae, T.; Schneewind, O. Allelic replacement in *Staphylococcus aureus* with inducible counter-selection. *Plasmid* **2006**, *55*, 58–63.
3. Pinho, M.G.; Errington, J. A *divIVA* null mutant of *Staphylococcus aureus* undergoes normal cell division. *FEMS Microbiol. Lett.* **2004**, *240*, 145–149.
4. ImageJ. Available online: http://rsb.info.nih.gov/ij/index.html (accessed on 24 December 2012).
5. Duthie, E.S.; Lorenz, L.L. Staphylococcal coagulase; mode of action and antigenicity. *J. Gen. Microbiol.* **1952**, *6*, 95–107.
6. Kreiswirth, B.N.; Lofdahl, S.; Betley, M.J.; O'Reilly, M.; Schlievert, P.M.; Bergdoll, M.S.; Novick, R.P. The toxic shock syndrome exotoxin structural gene is not detectably transmitted by a prophage. *Nature* **1983**, *305*, 709–712.
7. Henze, U.; Sidow, T.; Wecke, J.; Labischinski, H.; Berger-Bächi, B. Influence of *femB* on methicillin resistance and peptidoglycan metabolism in *Staphylococcus aureus*. *J. Bacteriol.* **1993**, *175*, 1612–1620.
8. Miroux, B.; Walker, J.E. Over-production of proteins in *Escherichia coli*: Mutant hosts that allow synthesis of some membrane proteins and globular proteins at high levels. *J. Mol. Biol.* **1996**, *260*, 289–298.
9. Lee, E.; Manoil, C. Mutations eliminating the protein export function of a membrane- spanning sequence. *J. Biol. Chem.* **1994**, *269*, 28822–28828.
10. Karimova, G.; Pidoux, J.; Ullmann, A.; Ladant, D. A bacterial two-hybrid system based on a reconstituted signal transduction pathway. *Proc. Natl. Acad. Sci. USA* **1998**, *95*, 5752–5756.
11. Rohrer, S.; Berger-Bächi, B. Application of a bacterial two-hybrid system for the analysis of protein-protein interactions between FemABX family proteins. *Microbiology* **2003**, *149*, 2733–2738.

12. Drew, D.; Sjöstrand, D.; Nilsson, J.; Urbig, T.; Chin, C.N.; de Gier, J.W.; von Heijne, G. Rapid topology mapping of *Escherichia coli* inner-membrane proteins by prediction and PhoA/GFP fusion analysis. *Proc. Natl. Acad. Sci. USA* **2002**, *99*, 2690–2695.
13. Karimova, G.; Dautin, N.; Ladant, D. Interaction network among *Escherichia coli* membrane proteins involved in cell division as revealed by bacterial two-hybrid analysis. *J. Bacteriol.* **2005**, *187*, 2233–2243.
14. Reed, P.; Veiga, H.; Jorge, A.M.; Terrak, M.; Pinho, M.G. Monofunctional transglycosylases are not essential for *Staphylococcus aureus* cell wall synthesis. *J. Bacteriol.* **2011**, *193*, 2549–2556.
15. Rohrer, S. Studies on members of the FemABX protein family in *Staphylococcus aureus*. Ph.D. Thesis, Swiss Federal Institute of Technology, Zürich, Switzerland, 2002; p. 152.

© 2013 by the authors; licensee MDPI, Basel, Switzerland. This article is an open access article distributed under the terms and conditions of the Creative Commons Attribution license (http://creativecommons.org/licenses/by/3.0/).
